# A Role for PP1/NIPP1 in Steering Migration of Human Cancer Cells

**DOI:** 10.1371/journal.pone.0040769

**Published:** 2012-07-16

**Authors:** Cristina Martin-Granados, Alan R. Prescott, Nele Van Dessel, Aleyde Van Eynde, Miguel Arocena, Izabela P. Klaska, Janina Görnemann, Monique Beullens, Mathieu Bollen, John V. Forrester, Colin D. McCaig

**Affiliations:** 1 From the Institute of Medical Sciences, University of Aberdeen, Aberdeen, Scotland, United Kingdom; 2 College of Life Sciences, University of Dundee, Dundee, Scotland, United Kingdom; 3 Laboratory of Biosignaling & Therapeutics, Department of Cellular and Molecular Medicine, University of Leuven, Leuven, Belgium; King’s College London, United Kingdom

## Abstract

Electrical gradients are present in many developing and regenerating tissues and around tumours. Mimicking endogenous electric fields *in vitro* has profound effects on the behaviour of many cell types. Intriguingly, specific cell types migrate cathodally, others anodally and some polarise with their long axis perpendicular to the electric vector. These striking phenomena are likely to have *in vivo* relevance since one of the determining factors during cancer metastasis is the ability to switch between attractive and repulsive migration in response to extracellular guidance stimuli. We present evidence that the cervical cancer cell line HeLa migrates cathodally in a direct current electric field of physiological intensity, while the strongly metastatic prostate cancer cell line PC-3-M migrates anodally. Notably, genetic disruption of protein serine/threonine phosphatase-1 (PP1) and its regulator NIPP1 decrease directional migration in these cell lines. Conversely, the inducible expression of NIPP1 switched the directional response of HeLa cells from cathodal to slightly anodal in a PP1-dependent manner. Remarkably, induction of a hyperactive PP1/NIPP1 holoenzyme, further shifted directional migration towards the anode. We show that PP1 association with NIPP1 upregulates signalling by the GTPase Cdc42 and demonstrate that pharmacological inhibition of Cdc42 in cells overexpressing NIPP1 recovered cathodal migration. Taken together, we provide the first evidence for regulation of directional cell migration by NIPP1. In addition, we identify PP1/NIPP1 as a novel molecular compass that controls directed cell migration via upregulation of Cdc42 signalling and suggest a way by which PP1/NIPP1 may contribute to the migratory properties of cancer cells.

## Introduction

Cell migration plays a pivotal role in many processes such as embryonic development and wound repair and mis-regulated signalling responses to migratory cues can induce pathologies such as tumour metastasis, inflammation and epilepsy [1]–[4]. Epithelial, endothelial, neuronal and immune cells, amongst others, are exposed to a variety of stimuli that direct cell migration. In addition to the more widely recognised chemical signals, such as growth factors and cytokines, endogenously generated electric fields (EF) of ionic nature have been measured around injured tissues, sites of inflammation and tumours [5]–[10]. These electrical signals can act as directional guidance cues during wound healing, embryonic development and tumorigenesis [11], therefore deciphering the molecular mechanisms behind the cellular responses to EF is of great importance. Applying a steady, direct current (DC) EF to cells and tissues *in vitro* mimics the effects of an endogenous EF [12] and this has identified a number of cell surface receptors, phosphorylation signalling proteins and second messengers that transduce electrical signals. For instance, epidermal growth factor receptor (EGFR) and integrins are amongst the first sensors of the electrical signals in several cell types. EGFRs translocate within the plane of the lipid bilayer to accumulate at the cathodal, apical side of cells. For keratinocytes and corneal epithelial cells this occurs within 5–10 min of EF exposure [13], [14]. As a consequence, EGF signalling becomes polarised, causing greater cathodal activation of ERK1/2, downstream cathodal polymerization of F-actin and directed migration [13]–[15]. Similar findings have been reported to underpin cathodal electrotaxis of embryonic and adult neural progenitor cells [16]. In addition, integrins α5 and α5ß1 redistribute and aggregate cathodally on fibroblasts migrating cathodally, as does β1 integrin in epithelial cells [17], [18]. Moreover, depletion of ß4 integrin or the addition of an anti-integrin β1 subunit antibody suppresses EF-directed migration [18], [19].

The role of protein tyrosine (Tyr) kinases in migration has been well studied, whereas the contribution of protein phosphatases has begun to be appreciated only recently [20]. In fact, the only phosphatase known to be involved in electrotaxis is the lipid phosphatase ‘phosphatase tensin homolog deleted on chromosome ten’ (PTEN) [7].

Protein serine/threonine (Ser/Thr) phosphatase-1 (PP1) is one of the most highly conserved enzymes known and plays a central role in a range of cellular processes including protein synthesis, RNA splicing, cell-cycle progression and glycogen metabolism [21], [22]. A large array of regulatory subunits associates with the PP1 catalytic subunit to determine its cellular localization and substrate specificity, mediating the control of these many physiological processes via PP1 holoenzymes [22]–[24]. NIPP1 (nuclear inhibitor of protein phosphatase 1) is a highly conserved and ubiquitously expressed protein that was initially characterized as a PP1 inhibitor [25]–[27]. NIPP1 serves as a kind of scaffold protein around which a variety of proteins such as phosphatases, kinases, splicing factors and chromatin modifiers gather functionally. NIPP1 contains two major PP1-interaction sites that reside in the central and C-terminal domains, among them the amino acid residues 200–203, which represent a RVxF-type PP1 docking site. More recent evidence suggests that the effects of NIPP1 on PP1 are substrate dependent: it potently blocks the dephosphorylation of many PP1 substrates but promotes the dephosphorylation of substrates that are recruited via its ForkHead Associated (FHA) domain [28]. Interestingly, PP1 bound to overexpressed wild-type NIPP1 (W.T-NIPP1) is highly phosphorylated at Thr-320, a mark which inactivates PP1, whereas PP1 bound to a C-terminus truncated NIPP1 protein (ΔC-NIPP1) is less phosphorylated at Thr-320, which is indicative for a hyperactive PP1/NIPP1 holoenzyme [28].

A role for PP1 as a regulator of cell polarity and migration is beginning to emerge. PP1 interacts with several proteins that regulate the actin cytoskeleton and contributes to the formation of cellular protrusions and adhesions [24]. Moreover, a very recent report has identified a functional role for PP1 in controlling enteric nerve cell migration [29]. Here, we investigated whether PP1 and NIPP1 levels regulate motility and directional migration of the cervical cancer-derived HeLa cell line. Further, we explored the contribution of NIPP1-associated PP1 to directional migration by using HeLa Tet-Off (HTO) cells that were engineered to inducibly express W.T-NIPP1, C-terminus truncated NIPP1 (ΔC-NIPP1) or a PP1-binding mutant of NIPP1 (mNIPP1) [28], [30]. We used a DC electric field (EF) as a readily tractable guidance cue known to control directed cell migration of normal and tumour cells [31], [32]. Here, we demonstrate that PP1 and NIPP1 levels are required for optimal random motility of single HeLa cells and for directed migration in response to a DC EF. We confirm that NIPP1 levels are required for directional cell migration by testing electrotaxis of the highly metastatic prostate cancer-derived cell line PC-3-M. Further, we demonstrate that binding of PP1 to NIPP1 functions as a compass which controls the direction in which cells migrate via regulating the expression of integrin and growth factor receptors, and Cdc42 GTPase activity. These results identify a functional role for NIPP1 in cell migration and uncover PP1/NIPP1 as the first protein Ser/Thr phosphatase complex controlling the directional response of cells to electrical guidance cues.

## Results

### PP1 and NIPP1 are Required for Random and Directional Migration of HeLa and PC-3-M Cells in Response to Electrical Guidance Cues

**Figure 1:**
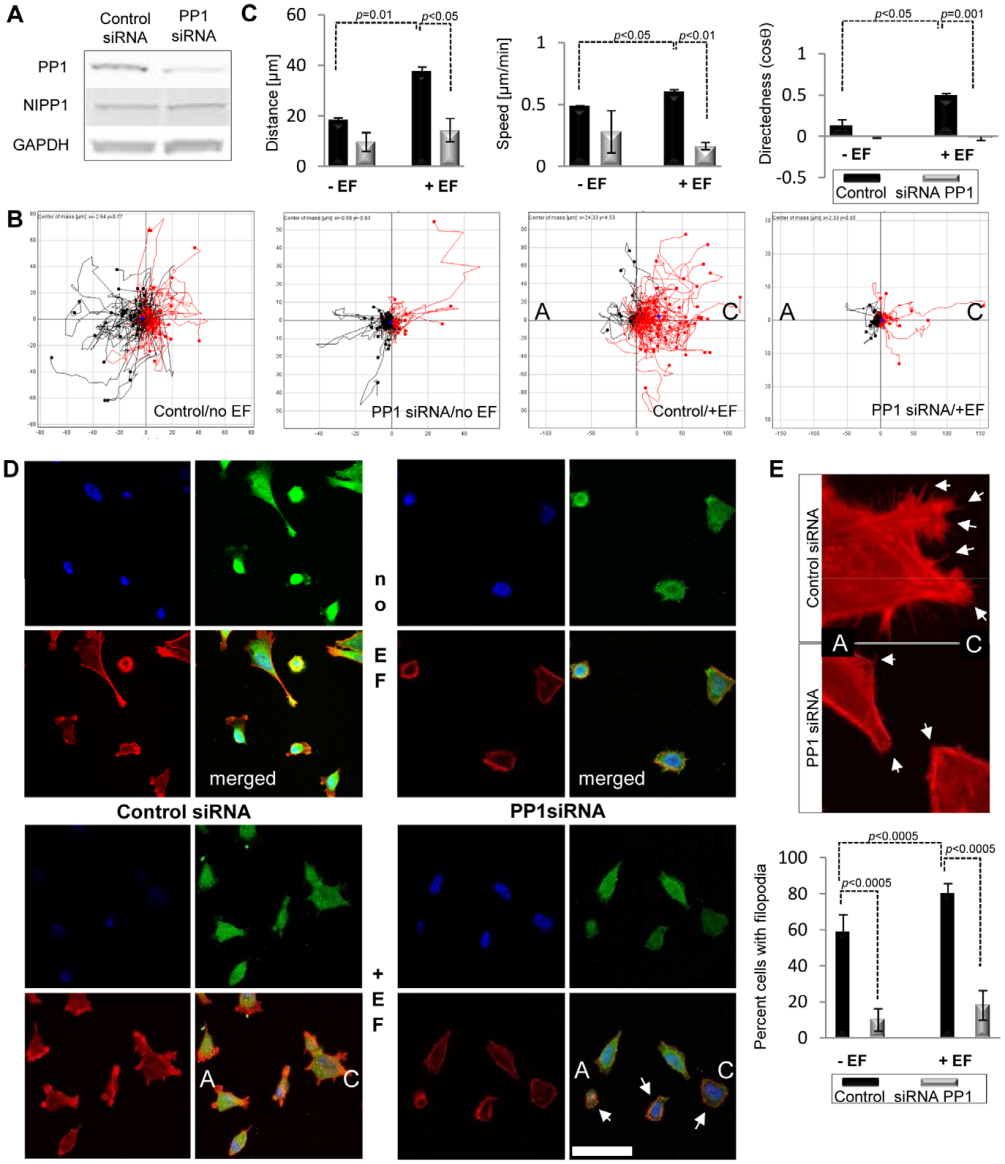
PP1 loss impairs electrotaxis in HeLa cells. **A.** Treatment of parental HeLa Tet-Off (HTO) cells with siRNA strongly depletes PP1 levels 48 h post transfection. Endogenous PP1 levels were visualized with PP1 antibodies that recognize all isoforms. **B.** Plot diagrams show that loss of PP1 impairs the ability of cells to migrate towards the cathode. Each line represents the migration trajectory of a single cell. The starting point for each cell migration track is at the origin. Cell tracks with end positions to the right appear in red (“C”, cathode) and those to the left appear in black (“A”, anode). EF-untreated cells were assayed as controls. Control siRNA cells migrate strongly towards the cathode; PP1 siRNA treated cells are unable to migrate in response to a DC EF. Scales show distance migrated in µm. **C.** PP1 depletion strongly reduces distance migrated, speed, and directedness in response to physiological DC EF. Error bars are S.E.M. *p* values for significant differences in distance, speed and directedness are shown. **D.** Localization of endogenous PP1 and distribution of filamentous-actin in control and PP1 depleted cells treated with DC EF. Endogenous PP1 levels were visualized with PP1 antibodies that recognize all isoforms (green) and polymerised actin was detected using rhodamine phalloidin (red). The nuclei have been stained with DAPI (blue). Arrows mark cells with a strong decrease in PP1 levels which correlate with defects in the formation of actin rich protrusions. Representative images are shown. Scale bar is 50 µm. **E.** Numbers of cells with filopodia were quantified by counting 100 cells. Error bars are S.E.M. *p* values for significant differences are shown. Images show a detail of cell protrusions in control siRNA and PP1 siRNA cells. Arrows mark numerous filopodia in control cells and outline areas with a major lack of filopodia at the cell edges in PP1 siRNA cells.

A very recent study has shown that treatment of enteric neural crest cells with okadaic acid, an inhibitor of protein phosphatases 1 and 2A, induces undirected cell protrusions and random cell movements [29]. Hence, we investigated a potential role for PP1 in regulating directional migration of cervical epithelium carcinoma-derived HeLa Tet-Off (HTO) cells in response to electrical guidance cues. For this, we tested the effect of previously validated siRNAs targeting all three PP1 isoforms on random motility of single cells and on the directed migratory response of cells to an applied EF (electrotaxis) [33]. PP1 protein levels were reduced by 85% after 48 h of transfection (Fig. 1A). In the absence of an EF, both control and PP1 knockdown (KD) cells migrated randomly (Fig. 1B). When a DC EF was applied, 82% ±5 of control siRNA cells migrated cathodally (red, to the right) (Fig. 1B; see video S1). EF treatment increased the distance migrated, the speed of migration and the directedness of control siRNA cells (Fig. 1C). However, PP1 depletion completely impaired electrotaxis, 57% ±2 of PP1 siRNA cells migrated cathodally (red, right) and 43% ±2 anodally (black, left) (Fig. 1B,C; see video S2). Moreover, we observed that cells depleted in PP1 displayed less cellular protrusions and more stress fibers compared to control siRNA cells (Fig. 1D). In particular, loss of PP1 decreased filopodia formation in untreated and EF-treated cells (Fig. 1E).

**Figure 2:**
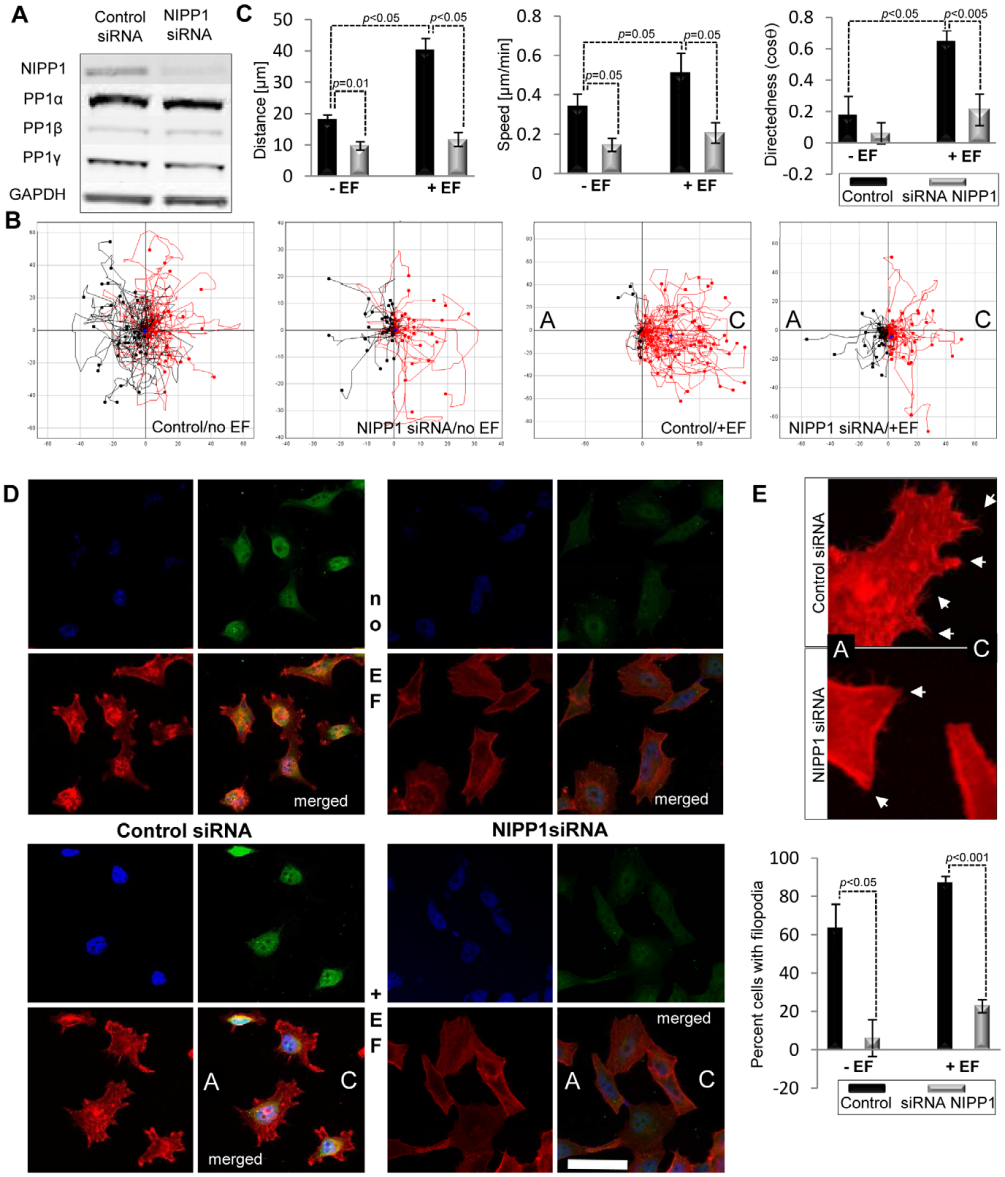
Loss of the PP1 interactor NIPP1 impairs the electrotactic response of HeLa cells. **A.** Treatment of parental HeLa Tet-Off (HTO) cells with siRNA strongly depletes NIPP1 levels 48 h post transfection. Cell lysates were analysed by SDS/PAGE and immunoblotting. Bands corresponding to all PP1 isoforms were detected and GAPDH was used as loading control. **B.** Plot diagrams show that loss of NIPP1 impairs the ability of cells to migrate towards the cathode. Control siRNA cells migrate strongly towards the cathode; NIPP1 siRNA treated cells show a much reduced cathodal response. Scales show distance migrated in µm. Scales are different between diagrams in order to include the tracks of every cell assayed. **C.** NIPP1 depletion strongly reduces distance migrated, speed, and directedness in response to physiological DC EF. Data are from at least three experiments. Error bars are S.E.M. *p* values for significant differences in distance, speed and directedness are shown. **D.** Localization of endogenous NIPP1 and distribution of filamentous-actin in control and NIPP1 depleted cells treated with DC EF. Endogenous NIPP1 levels were recognized with a rabbit anti-NIPP1 antibody (green) and polymerised actin was detected using rhodamine phalloidin (red). Nuclei are stained with DAPI (blue). NIPP1 localizes to the nucleus in EF-treated and untreated cells and its levels are depleted by siRNA. Scale bar is 50 µm. **E.** Numbers of cells with filopodia were quantified by counting 100 cells. Error bars are S.E.M. *p* values for significant differences are shown. Images show a detail of cell protrusions in control siRNA and NIPP1si RNA cells. Arrows mark numerous filopodia in control cells and outline areas with a major lack of filopodia at the cell edges in NIPP1 siRNA cells.

Further, we investigated a possible regulatory role for the PP1 interactor NIPP1 in the formation of actin protrusions and in random and directional migration of HTO cells. Firstly, we examined whether NIPP1 is required for migration by testing the effect of previously validated siRNAs targeting NIPP1 [34]. NIPP1 protein levels were reduced by 80% after 48 h of transfection (Fig. 2A). In the absence of an EF, both control and NIPP1 knockdown (KD) cells migrated randomly (Fig. 2B). In the presence of an EF, control cells showed strong cathodal migration; 87% ±4 of control siRNA cells migrated cathodally (red, right) and 13% ±4 anodally (black, left) (Fig. 2B; see video S3). However, NIPP1 KD cells showed a much blunted cathodal migration, 57% ±3 of NIPP1 siRNA cells migrated cathodally (red, right) and 43% ±3 anodally (black, left) (Fig. 2B,C and see video S4). Moreover, in the absence of an EF a two-fold decrease in the speed and therefore the distance of cell migration was seen in NIPP1 siRNA cells compared to control siRNA treated cells (Fig. 2C). The reduced speed and distance of migration caused by loss of NIPP1 was even greater in cells exposed to an EF which showed a four-fold decrease in cell migration and over a two-fold decrease in speed of migration compared to EF-treated control siRNA cells (Fig. 2C). Consistent with previous reports, the DC EF promoted actin polymerization and formation of actin-rich cell protrusions in control HTO cells (Fig. 2D). However, NIPP1 KD cells had fewer cell protrusions (Fig. 2D). In particular, the ability to form filopodia in NIPP1 KD cells was compromised severely in untreated and EF-treated cells (Fig. 2E).

**Figure 3:**
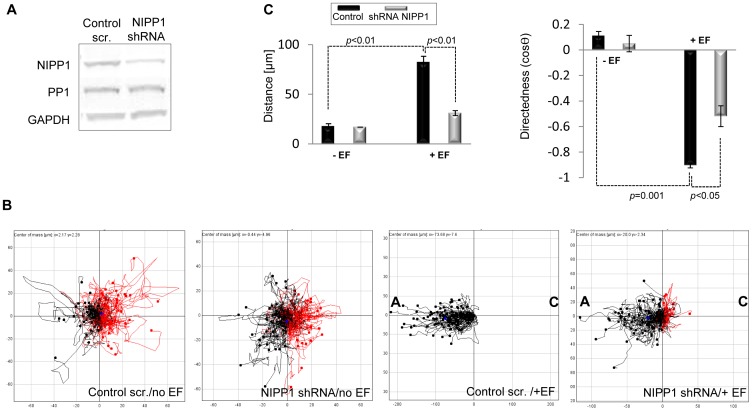
Loss of the PP1 interactor NIPP1 impairs the electrotactic response of PC-3-M cells. **A.** Treatment of PC-3-M cells with IPTG induces NIPP1 depletion. Cell lysates were analysed by SDS/PAGE and immunoblotting. Bands corresponding to the PP1 isoforms were detected and GAPDH was used as loading control. **B.** Plot diagrams show that loss of NIPP1 impairs the ability of PC-3-M cells to migrate anodally. Migration trajectories were tracked for three hours. The starting point for each cell migration track is at the origin. Cell tracks with end positions to the right appear in red and those to the left appear in black. Cathode is marked as “C” and anode as “A” when a DC EF is applied to cells. Control scrambled PC-3-M cells migrate strongly anodally (negative directedness value); cells expressing shRNA targeting NIPP1 show a much reduced anodal response. Scales show distance migrated in µm. Scales are different between diagrams in order to include the tracks of every cell assayed. **C.** NIPP1 depletion strongly reduced distance migrated and directedness in response to physiological DC EF. Data are from at least three experiments. Error bars are S.E.M. *p* values for significant differences in distance, speed and directedness are shown.

To further validate our data and to rule out possible off-target effects of the siRNA targeting NIPP1 we also examined the electrotactic response of the highly metastatic human prostate cancer cell line, PC-3-M, depleted in NIPP1 levels via expression of a shRNA targeting NIPP1 after IPTG treatment. NIPP1 levels were reduced by about 70% after 5 days of IPTG treatment (Fig. 3A). We show for the first time that PC-3-M cells display a very robust electrotactic response towards the anode as indicated by a strongly negative directedness of −0.9 (Fig. 3B,C and see video S5) and that loss of NIPP1 strongly reduces the directional response of these cells to a DC EF (Fig. 3B,C and see video S6).

Collectively, these data show that both PP1 and NIPP1 are required for the directional migratory response of HeLa and PC-3-M cells to a DC EF.

### PP1/NIPP1 Controls Directional Cell Migration

**Figure 4:**
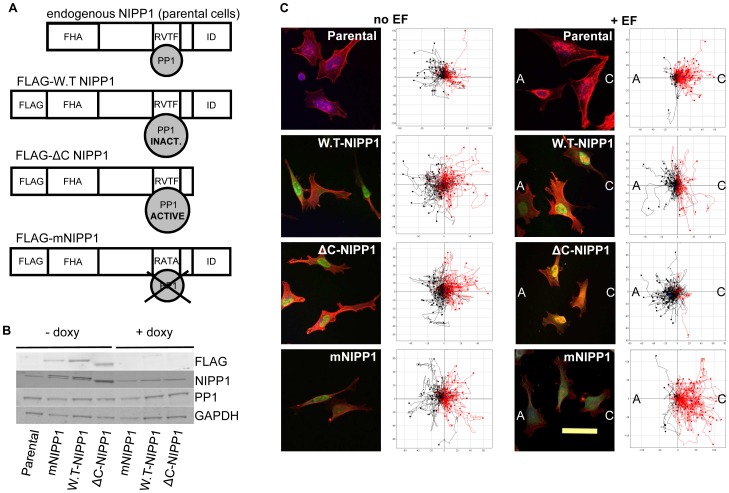
NIPP1 expression in HTO cells and control of EF-induced directional migration via its binding to PP1. **A.** Cartoon of endogenous NIPP1 and the different FLAG-tagged NIPP1 variants expressed after doxycyclin removal in HeLa Tet-Off (HTO) cell lines. All three NIPP1 variants have a forkhead associated domain (FHA). The consensus PP1-binding sequence, RVTF in W.T-NIPP1 has been mutated to RATA in the FLAG-mNIPP1 variant. The C-terminal auto-inhibitory (ID) domain is not included in the FLAG tagged ΔC-NIPP1 protein, resulting in the expression of a constitutively active PP1/NIPP1 holoenzyme. **B.** Expression of NIPP1 variants confirmed by Western blotting after removal of doxycyclin. Cell lysates were analysed by SDS/PAGE and immunoblotting. Bands corresponding to the PP1 isoforms were detected and GAPDH was used as loading control. **C.** NIPP1 expression and localization in the HTO cells was confirmed by ICC in EF-treated and untreated HTO cells. Anti-FLAG antibody and rhodamine phalloidin have been used to detect the FLAG-tagged NIPP1 variants (green) and F-actin (red). The nuclei were stained with DAPI. Overexpressed NIPP1 localizes to the nucleus in EF-treated and untreated cells. Scale bar is 50 µm. Plot diagrams show that an EF of physiological strength (200 mV/mm) induced distinct migratory responses in the HTO cells expressing different NIPP1 variants. EF-untreated HTO cells are shown as controls. Migration trajectories were tracked for three hours in the absence and presence of EF. Each cell’s position at 0 h is positioned at the origin (0, 0). Cells whose end position is to the right are coloured red and those to the left appear in black. Cathode is marked as “C” and anode is marked as “A” when DC EF is applied to cells. Scales show distance migrated in µm. Note that scales are different among diagrams in order to include the tracks of every cell assayed.

Next, we took a reverse approach and explored the effect of the overexpression of NIPP1 and its binding to PP1 on EF-induced directional migration. For this, we used previously characterized HeLa Tet-Off (HTO) cell lines that express three different NIPP1 variants in the absence of doxycylin (Fig. 4A) [28], [30]. Three days after doxycyclin removal from the medium, the expression of the NIPP1 variants was evaluated by Western blotting (Fig. 4B). These variants included FLAG-tagged W.T-NIPP1, which is associated with (partially) inactive PP1, C-terminally nicked FLAG-NIPP1 (ΔC-NIPP1) which is complexed to constitutively active PP1 and a point mutant (mNIPP1) that lacks a functional RVxF-type PP1 binding motif and can therefore only marginally bind to PP1 (Fig. 4A).

Localization of the three different NIPP1 variants was examined by immunocytochemistry. Similar to endogenous NIPP1, FLAG-tagged W.T- and ΔC-NIPP1 were localized strongly to the nucleus in EF-treated and untreated cells (Fig. 4C, cell images). Perinuclear staining of FLAG-tagged PP1-binding mutant of NIPP1 (mNIPP1) could also be observed (Fig. 4C, cell images).

Next, we tested whether the association of PP1 with NIPP1 affects electrotaxis of HTO cells. Without the EF, cell migration was oriented randomly for all cell types (Fig. 4C, “no EF” plots). However, HTO cells expressing FLAG-tagged NIPP1 variants showed an array of different behaviors in an EF. 72% ±3 of parental HTO cells migrated cathodally (right) and 28% ±3 anodally (left) and displayed a directedness of 0.27±0.05 (Fig. 4C; see video S7). Overexpression of W.T-NIPP1 shifted the cathodal response to slightly anodal, with only 35%±12 of cells migrating cathodally and 65%±12 anodally, and a directedness of −0.12±0.05 (Fig. 4C; see video S8). Moreover, overexpression of ΔC-NIPP1 induced a strong shift in the directional response. Only 16%±5 of cells migrated cathodally with a remarkable 84%±5 of cells migrating anodally giving a strongly reversed directedness of −0.55±0.04 (Fig. 4C; see video S9). Interestingly, overexpression of mNIPP1 did not affect cathodal migration and the cells behaved similarly to parental HTO cells; 80%±13 migrated cathodally and 20%±13 anodally, and displayed a strong cathodal directedness of 0.52±0.1 (Fig. 4C; see video S10).

These results show that control of directional migration by NIPP1 depends on its association with PP1. When NIPP1 was overexpressed and able to bind PP1, the cathodal migration shifted to slightly anodal. Moreover, induction of a constitutively active PP1/NIPP1 holoenzyme induced an even stronger anodal response. However, the PP1-binding mutant of NIPP1 caused cathodal migration, similar to parental cells.

### PP1/NIPP1 Controls Centrosome Positioning during Migration

**Figure 5:**
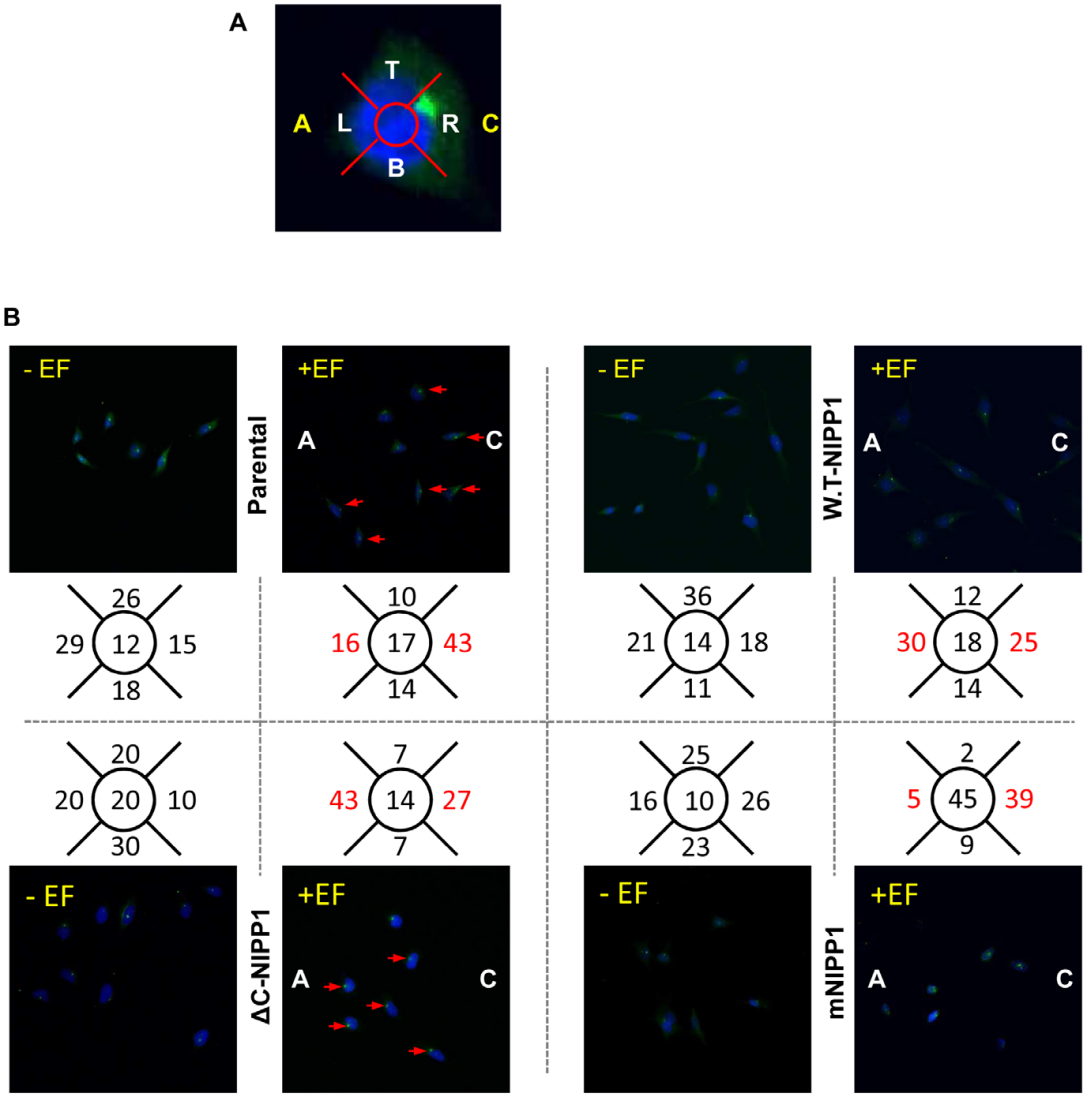
Centrosome polarization in the HTO cells mirrors directional migration in EF. **A.** A DC EF polarizes centrosomes to the cathode in parental HTO cells as seen by counting the cells in 5 regions, top (t), Right (cathode in EF-treated cells), bottom (b), left (anode in EF-treated cells) and centre of the nucleus (marked as a white dot). **B.** Parental cells and mNIPP1 cells position their centrosomes cathodally in an EF, whereas overexpression of W.T-NIPP1 disrupts cathodal centrosomal polarisation and overexpression of ΔC-NIPP1 shifts cathodal polarisation of centrosomes to anodal. 100 cells were counted in each case and results are expressed as percentages.

A correlation between the position of the centrosome and the direction of cell migration has been observed in several cell types [35]. In many cases the centrosome is located behind the leading edge and in front of the nucleus. Therefore, we next aimed to corroborate the results obtained from measuring the directional response of the HTO cells overexpressing FLAG-tagged NIPP1 variants by exploring whether there was a correlation between the position of the centrosome and the direction of cell movement in the HTO cells. In HTO cells (no EF) centrosomes were positioned randomly (Fig. 5). Centrosomes of parental cells in an EF polarized cathodally (Polarization index (PI) = 0.46 (see Experimental Procedures); Fig. 5). However, cells overexpressing W.T-NIPP1 displayed nearly the same distribution of centrosomes towards the cathode and anode (PI = −0.09; Fig. 5). Disruption of EF-induced cathodal centrosomal polarisation in cells overexpressing W.T-NIPP1 was dependent on PP1 binding to NIPP1 because cells overexpressing mNIPP1 polarised their centrosomes towards the cathode (PI = 0.77), as did parental cells (PI = 0.46; Fig. 5). Intriguingly, induction of a constitutively active PP1/NIPP1 holoenzyme induced both a strong anodal polarisation of centrosomes (PI = −0.23) and strong anodal migration of cells (Figs. 4C, 5). These findings demonstrate that the positioning of the centrosome during migration mirrors the directional migration in HeLa cells. Most significantly, this data indicates that association of PP1 with NIPP1 controls the switch to anodal centrosome polarisation and anodal migration, probably reflecting engagement of similar cytoskeletal machinery in both processes.

### Inhibition of Cdc42 Reverses the NIPP1-induced Anodal Migration and Centrosomal Polarization

The effects of NIPP1 on the formation of filopodia (Fig. 2), directional cell migration (Fig. 4C) and centrosome positioning in a physiological EF (Fig. 5) are dependent on PP1. Interestingly, a genome-wide profiling of the HTO cells uncovered that NIPP1 also affects the expression of numerous genes in a PP1-dependent manner [30]. It is well established that the GTPase Cdc42 controls filopodial extension and centrosome positioning in migrating cells [36]–[38], and that the directional migration of corneal epithelial cells in response to a DC EF is controlled by a Cdc42/Rho switch [39]. Collectively, these data lead to the enticing hypothesis that the NIPP1-induced anodal polarisation is mediated by signalling through Cdc42. To test this notion, we first analysed the list of genes that are significantly upregulated by the overexpression of W.T-NIPP1 or ΔC-NIPP1, but not by mNIPP1, all compared to the parental HTO cell line [30] and unpublished data (see materials and methods). Interestingly, we found 24 genes that are involved in cytoskeletal dynamics, cell-matrix interactions and the Cdc42 pathway, and are activated by overexpression of W.T-NIPP1 or ΔC-NIPP1, but not by mNIPP1 (Table 1).

**Table 1:** List of genes from the Cdc42 pathway that are significantly upregulated by the overexpression of W.T-NIPP1 (WT) or ΔC-NIPP1 (ΔC), but not by mNIPP1 (m), in the HTO cells and compared to parental HTO cells.

Gene	Fold change				Class
	Parental	WT	ΔC	mNIPP1	
**DDR2**	1	2	3	1	Receptor tyrosine kinase
**EGFR**	1	1	2	1	Receptor tyrosine kinase
**EPHA2**	1	3	4	1	Receptor tyrosine kinase
**EPHA4**	1	2	1	1	Receptor tyrosine kinase
**EPHB2**	1	2	1	1	Receptor tyrosine kinase
**FGFR1**	1	2	2	1	Receptor tyrosine kinase
**ITGA1**	1	1	4	1	Integrin receptors
**ITGA2**	1	2	1	1	Integrin receptors
**ITGA5**	1	2	1	1	Integrin receptors
**ITGA6**	1	4	4	1	Integrin receptors
**ITGA11**	1	1	6	1	Integrin receptors
**ITGAV**	1	2	2	1	Integrin receptors
**ITGB1**	1	1	2	1	Integrin receptors
**ITGB2**	1	6	4	1	Integrin receptors
**ITGB3**	1	4	4	1	Integrin receptors
**ITGB4**	1	1	2	1	Integrin receptors
**ITGB5**	1	2	2	1	Integrin receptors
**CFL2**	1	1	3	1	Actin remodeling protein
**ACTR3**	1	1	2	1	Actin remodeling protein
**IQGAP1**	1	1	2	1	Ras GTPase-activating-like protein
**JUN**	1	2	2	1	Transcription factor
**ACTA1**	1	2	1	1	Cytoskeletal protein
**ACTA2**	1	32	108	1	Cytoskeletal protein
**ACTG2**	1	28	65	1	Cytoskeletal protein

**Figure 6:**
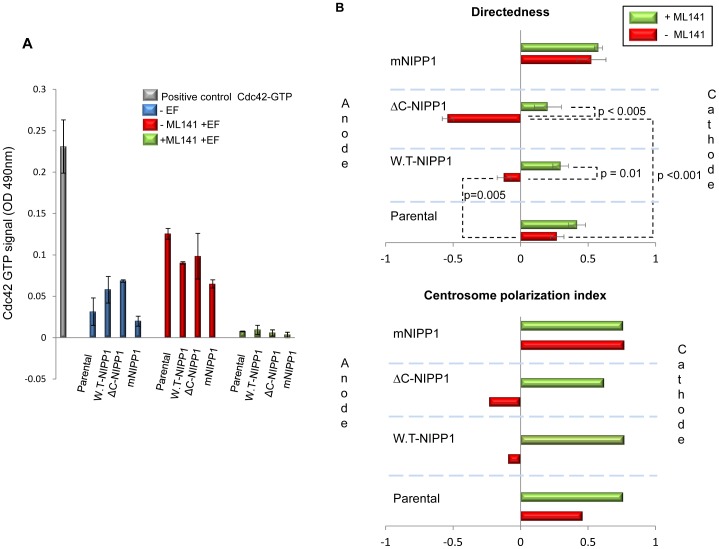
Effect of pharmacological inhibition of Cdc42-GTPase on the HTO cells. **A.** Effect of ML141 on Cdc42 GTPase activity in unstimulated cells cultured in complete medium and in EF-stimulated HTO cells overexpressing the FLAG-NIPP1 protein variants. Levels of Cdc42-GTP determined by G-LISA in parental, W.T-NIPP1, ΔC-NIPP1 and mRATA cells in the absence or presence of DC EF and in cells pre-treated with 10 µM of ML141 before electrical stimulation. *p* values parental to W.T-NIPP1 and parental to ΔC-NIPP1 in complete medium were 0.1 and 0.01, respectively; *p* values comparing samples in the absence and presence of ML141 were in all cases <0.01. **B.** Cdc42 inhibition rescues cathodal polarisation and this correlates with centrosome positioning. Directedness values for the migration of EF-treated cells incubated with ML141. Cdc42 inhibition rescues the positive cell directedness decreased by W.T-NIPP1 overexpression. The strongly negative directedness value displayed by ΔC-NIPP1 cells becomes closer to 0 when cells are pretreated with Cdc42 inhibitor. For simplification directedness values in the absence of EF of the parental, W.T-NIPP1, ΔC-NIPP1, and mNIPP1 with and without ML141 have not been included in the diagram. These were, without ML141, −0.07±0.04; 0.05±0.09; −0.08±0.05 and −0.01±0.04, respectively; with ML141 were −0.07±0.04; 0.09±0.05; −0.07±0.05 and −0.01±0.04, respectively. In the absence of EF values were in all cases very close to 0 and differences between the four lines were not statistically significant in any of the cases. Data was quantified from at least three experiments. Error bars are S.E.M. *p* values for significant differences in directedness are shown. Polarisation index of centrosomes calculated as explained in materials and methods. Polarisation index of W.T-NIPP1 and ΔC-NIPP1 cells becomes similar to the polarisation index of parental cells when cells are treated with the Cdc42 inhibitor ML141.

Next, we measured the Cdc42 GTPase activity in HTO cells in a physiological EF and verified whether the measured activity was inhibited by the specific and cell-permeable Cdc42 GTPase inhibitor ML141 (CID2950007) [40]. We found that a DC EF induces a small but significant increase in Cdc42 GTPase activity in all HTO cells (Fig. 6A; p<0.001) and that treatment of these cells with 10 µM ML141 completely abolished Cdc42 GTPase activity (p values comparing samples in the absence and presence of ML141 were in all cases <0.01). Interestingly, inhibition of Cdc42 did not affect cathodal migration of parental cells (directedness = 0.42±0.06; Fig. 6B; see video S11). However, the reversal in EF-directed migration by overexpression of W.T-NIPP1 was recovered by inhibition of Cdc42 (directedness = 0.29±0.05; Fig. 6B; see video S12). Similarly, EF exposed ΔC-NIPP1 cells, which migrate anodally, lost this response when Cdc42 was inhibited with ML141 and even displayed a moderate cathodal response (directedness = 0.2±0.1; Fig. 6B; see video S13). EF-stimulation of these cells (+ML141) promoted formation of stress fibers (data not shown) and induced cell spreading and ruffling together with blebbing of the actin cytoskeleton (data not shown). In many cases where strong ruffling was observed, cells became detached. An increase in the sub-G1 population (dead cells) in the ML141-pretreated ΔC-NIPP1 cells (determined by flow cytometry) may possibly account for the low migration and detachment observed in these cells. In contrast, ML141 did not cause a defect in viability of the parental, mNIPP1 and W.T-NIPP1 cells (Fig. S1).

Inhibition of Cdc42 GTPase in cells overexpressing NIPP1 but unable to bind PP1 (mNIPP1) did not affect their strong cathodal migration (directedness = 0.57±0.03; Fig. 6B; see video S14). These results clearly show that the switch in direction of EF-induced migration caused by overexpression of NIPP1 depends on the association of NIPP1 with PP1 and that it is mediated by Cdc42 GTPase activity.

We also investigated whether inhibition of Cdc42 in HTO cells could recover the polarisation of centrosomes towards the cathode in the W.T-NIPP1 and ΔC-NIPP1 cells. We demonstrate that Cdc42 inhibition increased the centrosomal PI of parental cells to levels comparable with those observed in mNIPP1 cells (PI = 0.76 in both cases), recovered the cathodal centrosomal polarisation in W.T-NIPP1 cells (PI = 0.59) and most strikingly, induced strong polarisation of centrosomes towards the cathode in ΔC-NIPP1 cells (PI = 0.62) (Fig. 6B). These findings indicate that EF-induced centrosomal polarisation towards the cathode is Cdc42 GTPase-independent. However, anodal polarisation of centrosomes observed in W.T-NIPP1 and ΔC-NIPP1 cells requires both its association with PP1 and Cdc42 GTPase activity.

## Discussion

Specifically, we found that both PP1 and NIPP1 positively regulate the formation of cell protrusions and that normal levels of the two proteins are required for optimal electrotaxis of cancer-derived cells. Further, we show that association of PP1 with NIPP1 controls directional migration and centrosome polarity.

### PP1 Binding to NIPP1 Controls Cell Polarity Via Cdc42-GTPase

Orientation of the microtubule organizing centre, or centrosome, towards the leading edge contributes to polarised migration by aiding microtubule growth into the lamella- and microtubule-mediated delivery of Golgi-derived vesicles to the leading edge, providing membrane and associated proteins for forward protrusion [41], [42]. In addition to establishing cell polarity, Cdc42 also regulates reorientation of the centrosome towards the leading edge [36], [38]. Indeed, many migrating cell types, including fibroblasts, neurons and macrophages, reorient the Golgi complex and the centrosome towards the leading edge during migration in 2D culture [43]–[45]. This reorientation also occurs during wound healing [46], application of a DC EF [47], shear stress [48], early development [49] and during antigen presentation to T-cells [50]. We have demonstrated here that in HeLa cells the centrosome is oriented towards the leading edge of the cell and that centrosomal polarisation in cells overexpressing the different NIPP1 variants mirrored exactly the direction of cell migration, i.e. cathodal centrosomal polarisation in parental and mNIPP1 cells, slightly anodal in W.T-cells and strongly anodal in ΔC-NIPP1 cells. These findings indicate that the association of PP1 with NIPP1 regulates cell polarity. We also show that Cdc42 GTPase activity is not essential for the establishment of cathodal centrosomal polarisation in the parental HTO cells, however it is required for anodal polarization in these cells.

### Upregulation of Cdc42 Signalling by PP1/NIPP1 Steers Directional Migration

Spatially and temporally coordinated activities of the small GTPases Rac, Cdc42 and Rho support polarized cell migration in a variety of cells [51]–[55]. Rho regulates stress fiber formation, motility and focal adhesions, while Rac is involved in lamellipodia and Cdc42 is more specifically involved in the formation of filopodia, the structures at the leading edge of the cell which “sense” guidance stimuli [56]. The contribution of Rho GTPases to polarized cell migration, initially observed during chemotaxis, has been extended to electrotaxis with the cathode as the attractant [57], [58]. In this model, activities of Rac and Cdc42 are elevated on the side of the cell facing the attractant and Rho activity is low. Conversely, on the side facing away from the attractant, Rho activity is high, with relatively low Cdc42 and Rac activities. Rho activation is downstream of Rac and both Rac and Rho are downstream of Cdc42 such that interplay between activation of these GTPases generates specific GTPase cascades with specific effects on the actin cytoskeleton and cell migration [59]. In agreement with this model, our data show that parental HTO cells with endogenous NIPP1 and PP1 levels migrate towards an attractive cue, i.e. the cathode. However, our findings in two cancer-derived cell lines are at odds with the idea that cathodal polarisation is driven by Cdc42, because treatment of parental HTO cells with the Cdc42 inhibitor, ML141, does not affect cathodal migration. In contrast, the anodal response induced in HeLa cells by W.T-NIPP1 and ΔC-NIPP1 overexpression required Cdc42-GTPase activity suggesting that Cdc42 acts as a downstream effector of PP1/NIPP1.

Here, we have shown that (1) NIPP1 is required for the formation of filopodia (Fig. 2), it controls directional cell migration (Fig. 4) and centrosome positioning (Fig. 5) in a physiological EF, in a PP1-dependent manner. Interestingly, genome-wide profiling of the HTO cells uncovered that NIPP1 affects the expression of numerous genes also in a PP1- dependent manner [30]. It is well established that Cdc42, controls filopodial extension and centrosome positioning in migrating cells [36]–[38]. Collectively, these data suggest that the NIPP1-induced anodal polarisation is mediated by Cdc42 signalling and therefore our research in this manuscript deals with proving this specific hypothesis.

In support of this mechanistic model by which NIPP1 association to PP1 controls the directional response via modulating Cdc42 activity, we show that PP1 binding to NIPP1 controls the expression of an array of genes implicated in Cdc42 signalling (Table 1) and that overexpression of PP1-associated NIPP1 increases Cdc42 GTPase activity when cultured in complete medium. We suggest that polarised distribution of different amounts and classes of membrane receptors in these cells may contribute to the transduction of the electric signal and to the variability in electrotaxis. In particular, high expression levels of integrin receptors may act cooperatively with EGFR to amplify the anodal response in ΔC-NIPP1 cells. In addition to the membrane receptors, Ephrin A and B receptors, which are upregulated in cells overexpressing PP1-associated NIPP1 and are implicated in the attraction/repulsion behaviour of cancer cells [60], [61] have been very recently suggested as sensors of electrical stimuli in highly metastatic lung cancer cells [62] and may contribute to the electrotactic properties of the cells tested here.

### Potential Physiological/Pathological Significance

**Figure 7:**
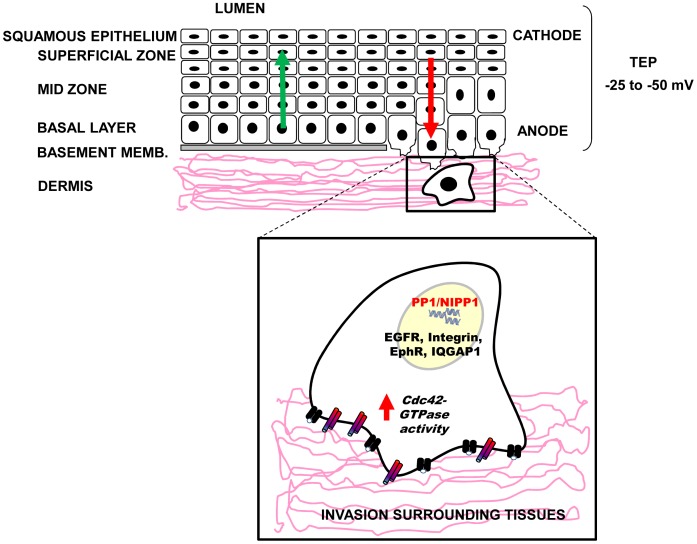
Cartoon showing the basic organization of the cervical epithelium and a mechanistic model to explain how PP1/NIPP1 may contribute to invasiveness of tumour cells. Cervical and vaginal epithelia have lumen potentials of about −25 to −50 mV [65], [66]. Such a lumen potential would correspond to a transepithelial voltage gradients of 1.7 V/cm (170 mV/mm). In these electrophysiological conditions cervical epithelial cells would migrate towards the lumen as they turn over the epithelial lining layer (green arrow). Upregulation of NIPP1 and its recruitment to PP1 would reverse migration into the lumen, encouraging invasion of the surrounding tissue (red arrow).

In addition to chemical gradients, electrical gradients exist across epithelia and in breast and prostate tumours [8], [10], [63]. Interestingly, electroimaging of mammary and cervical tissues has been used in clinical detection of malignancy [8], [9], [64]. Prostate epithelia, vaginal and cervical epithelium have lumen potentials of about −10 to −50 mV [65], [66]. Such a lumen potential would correspond to transepithelial voltage gradients of 5 V/cm in prostate epithelium [63] and 1.7 V/cm in cervical epithelium, assuming that the cellular thickness of the prostatic ducts is 20 µm and the cervical epithelium is 300 µm (Fig. 7). Similar to prostate cells described in a model suggested by Djamgoz et al., [63], under the above electrophysiological conditions cervical epithelial cells would migrate towards the lumen (cathodally). Such a voltage gradient in these tissues is comparable to the DC EF strengths used to induce electrotaxis in the present study. Alterations in the directional response of cells to electrical gradients have the potential to increase migration into the lumen or promote colonisation of surrounding tissues. Given that (1) NIPP1 is expressed in cervix and also in HeLa and PC-3-M cells derived from cervical and prostate tumours, respectively [67], (2) NIPP1 levels are tightly linked to malignant phenotype in tumours [68], (3) NIPP1 levels and its association to PP1 control directional cell migration, this suggests that an upregulation of PP1/NIPP1 is expected to reverse the “default” cathodal polarization (towards the lumen) and encourage invasion of the surrounding tissue (Fig. 7).

Taken together, we provide the first evidence for homeostatic regulation of cell migration by NIPP1. In addition, we identify the Ser/Thr phosphatase holoenzyme PP1/NIPP1 as a novel molecular compass that controls cell polarisation and directed cell migration in response to a physiological DC EF via upregulation of Cdc42 signalling. These intriguing findings suggest a Ser/Thr phosphatase-based mechanism for acquisition of a cell “metastatic” phenotype and pose novel opportunities for pharmacological interventions.

## Materials and Methods

### Chemical Reagents, Cell Cultures and Knockdown

Cell culture media and reagents were purchased from Invitrogen (Paisley, UK) and Clontech (CA, USA). The Cdc42 inhibitor ML141 was synthesized at Kansas University Specialized Chemistry Center. When ML141 was used, cell cultures were pre-treated with the inhibitor for 1 hour before applying the EF (also for controls without EF-stimulation). All experiments were conducted at least three times and performed within low passage of HeLa Tet-Off (HTO) cells expressing the different transgenes after doxycyclin removal [28], [30]. The culture conditions of HTO cells are described in Tanuma et al. and Van Dessel et al. [28], [30]. In all cases, expression of the FLAG-tagged NIPP1 variants was not higher than twice the levels of endogenous NIPP1. CO_2_-independent medium was used for experiments performed in room air.

SiRNA duplexes against three human PP1 isoforms, NIPP1 and scrambled control siRNAs were purchased from Dharmacon (Thermo Fisher Scientific, Tournai, Belgium) and Invitrogen (Paisley, UK), respectively. Sequences of the siRNAs against PP1 and NIPP1 are described in Van Dessel et al. and Qian et al., respectively [30], [33]. NIPP1 knockdown was performed in parental HTO cells using Lipofectamine RNAiMAX reagent (Invitrogen, Paisley, UK) and were analyzed after 48–72 h, as described in Nuytten et al. [34].

PC-3-M-Luc cells (Xenogen Corporation, CA, US) were transduced with pLKO_IPTG_1xLacO (Sigma-Aldrich, Dorset, UK) containing shRNA targeting PPP1R8 (TRC904-218076: TCCCACTTTCTAGGATCATTT) or non-target shRNA that does not target any human gene (Sigma-Aldrich, Dorset, UK) and selected with puromycin (Sigma-Aldrich, Dorset, UK). Expression of the shRNA was induced at concentration of 200 µM of isopropylthio-β-galactoside (IPTG) (Sigma-Aldrich, Dorset, UK) and optimal induction was shown after 3–5 days.

### Electrotaxis Experiments

Electrotaxis chambers of dimensions 4 cm×1 cm×0.5 mm were designed on laminin pre-coated plates as described previously [7]. HTO cells expressing the three NIPP1 variants and parental HTO cells were seeded in chambers at low density for 16–20 h. To prevent diffusion of electrode products into the cultures, DC EF were supplied through agar-salt bridges which connected silver/silver chloride electrodes via beakers of Steinberg’s solution to reservoirs of culture medium at either side of the chamber. Cell migration in the electrotaxis chambers was monitored with a Zeiss Axiovert 100 (Jena, Germany) microscope with a stage incubator controlling temperature at 37°C. DC EF (200 mV/mm) were applied for 3 hours to test cultures for electrotaxis.

### Analysis of Centrosome Polarization

After 1–2 h DC EF stimulation, HTO cells were fixed with 100% ice cold methanol for 15 min, permeabilized for 5 min with 0.3% Triton X-100 and blocked for 30 min in 10% donkey serum, 0.2% BSA in PBS. Cells were incubated overnight in the primary antibody solution (rabbit anti-pericentrin antibody from Santa Cruz Biotechnology, Heidelberg, Germany, diluted 1∶200 in 0.2% BSA in PBS). Cells were washed three times and incubated in a secondary antibody solution (donkey anti-rabbit secondary antibody Alexa fluor 488 in 0.2% BSA in PBS). Cells were washed three times and nuclei were stained with 1 µg/ml 4′,6-diamidino-2-phenylindole (DAPI) (Sigma-Aldrich, Dorset, UK) for 3 minutes. After washing, Hydromount (National diagnostics, USA) was added to the samples and coverslips of dimension 4 cm×0.8 cm were placed on the cells. Cells were imaged on a Zeiss LSM 700 confocal microscope (Zeiss, Jena, Germany). Cells were divided into five sections, i.e. centre, and 4×90° sectors top, bottom, left and right and centrosomes within these regions were counted. 100 cells were counted for each treatment and cell type and cells were scored as percentages. Cells with no EF supplied were regarded as control. A polarisation index (PI) also was calculated using the following formula:

PI  =  (% cells polarised cathodally - % cells polarised anodally)/(% cells polarised to the cathode + % cells polarised to the anode.

### G-LISA

Levels of Cdc42-GTP in the 4 HTO cell lines in the presence and absence of EF and Cdc42 inhibitor were measured using a G-LISA kit from Cytoskeleton (cat. Nr. BK127, Cytoskeleton, Inc., Denver, CO, USA) following manufacturer’s instructions and as described [40]. Electrical stimulation was carried out for 10 min in electrotaxis chambers placed in an incubator. Prior to application of DC EF, cells were treated for 1 h with 10 µM of ML141 inhibitor. Positive controls included Cdc42-GTP provided in the kit and negative controls included buffer-only samples.

### Western Blot Analysis

HTO cell cultures were lysed in lysis buffer (cat. Nr. C2978; Sigma-Aldrich, Dorset, UK) supplemented with protease and phosphatase inhibitors (Roche Diagnostics, Basel, Switzerland). 30 µg of total protein was used for immunoblot analysis following fractionation of proteins by sodium dodecyl sulphate polyacrylamide gel electrophoresis (SDS/PAGE) on 4–12% Bis–Tris gels (Novex, Invitrogen, Paisley, Scotland) and transference to nitrocellulose membranes (Invitrogen, Paisley, Scotland). Membranes were probed with rabbit anti-FLAG (Sigma-Aldrich, Dorset, UK) used at 1∶400, goat anti-PP1 antibodies (Santa Cruz Biotechnology, Heidelberg, Germany) used at 1∶500; rabbit anti-GAPDH (Abcam, Cambridge, U.K) used at 1∶2000. The mouse monoclonal NIPP1 antibody (mAb 15B8C11) was raised and screened against bacterially expressed full-length NIPP1 complexed to His-PP1 in 1∶1 ratio. These antibodies were purified and enriched from hybridoma medium by Protein A affinity chromatography and shown to recognize an epitope located in the central domain of NIPP1 (amino acids 143–224). Antibody binding was detected using donkey anti-goat, rabbit or mouse IgG[H + L] conjugated to an IRDye800 or 680 fluorophore (Rockland, Immunochemicals, Reading, UK) followed by analysis of the immunoblots using the Li-Cor Odyssey system.

### Immunocytochemistry

Bacterially expressed polyhistidine-tagged NIPP1 143–224 fragment was used to raise the antibodies in rabbits. These antibodies were affinity-purified on His-NIPP1-143-224 linked to CNBr-activated Sepharose 4B (GE Health care, Hertfordshire, UK).

After applying a DC EF for the indicated times, HTO cells were fixed in the chambers with 8% formaldehyde in PBS for 15 min. After removing the top of the chamber carefully, cells were washed with PBS permeabilized for 10 min with 1% NP-40, washed again and blocked for 30 min in 10% donkey serum or BSA in PBS. Cells were then incubated for 2 h in rabbit anti-FLAG antibody (Sigma-Aldrich, Dorset, UK) at 5 µg/ml in 0.2% BSA in PBS or overnight with the purified rabbit anti-NIPP1 antibodies diluted at 1∶125 in 1% BSA. Cells were washed three times and incubated for 45 min with Alexa-FluorTM488 and 594 conjugated to IgG (either anti-rabbit, goat or mouse) secondary antibodies (Molecular Probes) in 0.2% BSA in PBS and TRITC-Phalloidin (Sigma-Aldrich, Dorset, UK) used at 1∶1000. Cells were washed three times with 0.2% BSA in PBS and nuclei were stained with 1 µg/ml 4′,6-diamidino-2-phenylindole (DAPI) (Sigma-Aldrich, Dorset, UK) for 3 minutes. After washing, Hydromount was added to the cells before placing a coverslip of dimension 4 cm×0.8 cm to the 4 cm×1 cm on top. Chambers were left to dry overnight at 4°C. Cells were imaged on a Zeiss LSM 700 confocal microscope (Zeiss, Jena, Germany).

### Time-lapse Imaging and Quantification of Cell Migration

Time-lapse images were recorded every 5 min for 3 hours and migration trajectories of 100 cells were analyzed with ImageJ software and cell tracking and chemotaxis plugins. Migration directedness cosine θ, where θ is the angle between the EF vector and a straight line connecting the start and end position of a cell, was used as a parameter to indicate how directly a cell migrates in the presence and absence of DC EF [13], [69]. A cell moving exactly toward the cathode would have a directedness of 1; a cell moving perfectly along the field lines toward the anode would have a directedness of −1. Therefore, the average of directedness values of a population of cells gives an objective quantification of how directionally the cells have migrated. A group of cells migrating randomly would have an average directedness value of 0. Migration rate was analyzed with the following parameters. Speed of cell migration is the total length of the migration trajectory of a cell divided by the given period of time. The distance is the straight-line distance between the start and end positions of a cell.

### Gene Expression Analysis

The genome-wide expression profiling of the HTO cell lines stably expressing W.T-NIPP1 or mNIPP1 were described previously (Van Dessel et al, 2010) and the data are available at GEO under the accession number GSE19642. The genome-wide expression profiling of the ΔC-NIPP1 cell line was performed as the gene expression profiling of W.T-NIPP1 and mNIPP1 expressing HTO cell lines. The list of the genes that were significantly upregulated by the overexpression of W.T-NIPP1 or ΔC-NIPP1, but not by mNIPP1, were compared with a list of genes involved in the Cdc42 pathway. The latter list was composed of 144 genes, which were all linked to Cdc42 pathway based on Ingenuity Pathway Analysis (Ingenuity Systems Inc, USA) and the Human Protein Reference Database®.

### FACS Analysis

Cells were cultured for 3 days in the absence of doxycyclin. Adherent and floating cell fractions were collected separately and finally pooled together by gentle centrifugation. Cells were re-suspended in 1 ml of ice cold 70% ethanol (v/v) and fixed for at least 30 mins at room temperature. Cells were adjusted to approximately 0.5×10^5^ cells/ml and washed 2× in PBS +1% w/v BSA. Cells were then spun at 1000 g for 5 mins and re-suspended in 1 ml of staining buffer (50 µg/ml propidium iodide, 50 µg/ml ribonuclease A, 0.1% (v/v) in PBS) for 20 min at room temperature and protected from light and then analyzed by flow cytometry using BD FACSCalibur.

### Statistical Analysis

Experiments were performed at least three times and the data is the average of duplicate or triplicate determinations. Error bars show the standard error of the mean (S.E.M). Statistical analyses were performed using Student’s t-test.

## Supporting Information

Figure S1 FACS analysis showing the effect of ML141 in the cell cycle of parental, W.T-NIPP1, ΔC-NIPP1 and mNIPP1 HeLa Tet-Off cells. ML141 (1 h pre-treatment) does not have an effect on cell cycle of parental, W.T-NIPP1 and mNIPP1 cells, however the sub-G1 population of ΔC-NIPP1 cells appears increased. Three experiments were performed with similar results and a representative experiment is shown.
(TIF)

Video S1 Movie showing migration of control siRNA-treated parental HeLa Tet-Off cells in a physiological EF (cathode to the right, t = 3 h).
(MOV)

Video S2 Movie showing migration of PP1 siRNA-treated parental HeLa Tet-Off cells in a physiological EF (cathode to the right, t = 3 h).
(MOV)

Video S3 Movie showing migration of control siRNA-treated parental HeLa Tet-Off cells in a physiological EF (cathode to the right, t = 3 h).
(MOV)

Video S4 Movie showing migration of NIPP1 siRNA-treated parental HeLa Tet-Off cells in a physiological EF (cathode to the right, t = 3 h).
(MOV)

Video S5 Movie showing migration of control non-target PC-3-M cells in a physiological EF (cathode to the right, t = 3 h).
(MOV)

Video S6 Movie showing migration of NIPP1 shRNA PC-3-M cells in a physiological EF (cathode to the right, t = 3 h).
(MOV)

Video S7 Movie showing migration of parental HeLa Tet-Off cells in a physiological EF (cathode to the right, t = 3 h).
(MOV)

Video S8 Movie showing migration of W.T-NIPP1 HeLa Tet-Off cells in a physiological EF (cathode to the right, t = 3 h).
(MOV)

Video S9 Movie showing migration of ΔC-NIPP1 HeLa Tet-Off cells in a physiological EF (cathode to the right, t = 3 h).
(MOV)

Video S10 Movie showing migration of mNIPP1 HeLa Tet-Off cells in a physiological EF (cathode to the right, t = 3 h).
(MOV)

Video S11 Movie showing migration of ML141 pre-treated parental HeLa Tet-Off cells in a physiological EF (cathode to the right, t = 3 h).
(MOV)

Video S12 Movie showing migration of ML141 pre-treated W.T-NIPP1 HeLa Tet-Off cells in a physiological EF (cathode to the right, t = 3 h).
(MOV)

Video S13 Movie showing migration of ML141 pretreated ΔC-NIPP1 HeLa Tet-Off cells in a physiological EF (cathode to the right, t = 3 h).
(MOV)

Video S14 Movie showing migration of ML141 pretreated mNIPP1 cells in a physiological EF (cathode to the right, t = 3 h).
(MOV)
