# Supplementary figures and images for: A Role for PP1/NIPP1 in Steering Migration of Human Cancer Cells
Source: PLoS One. 2012 Jul 16;7(7):e40769. doi: 10.1371/journal.pone.0040769 (PMC3397927; doi:10.1371/journal.pone.0040769)

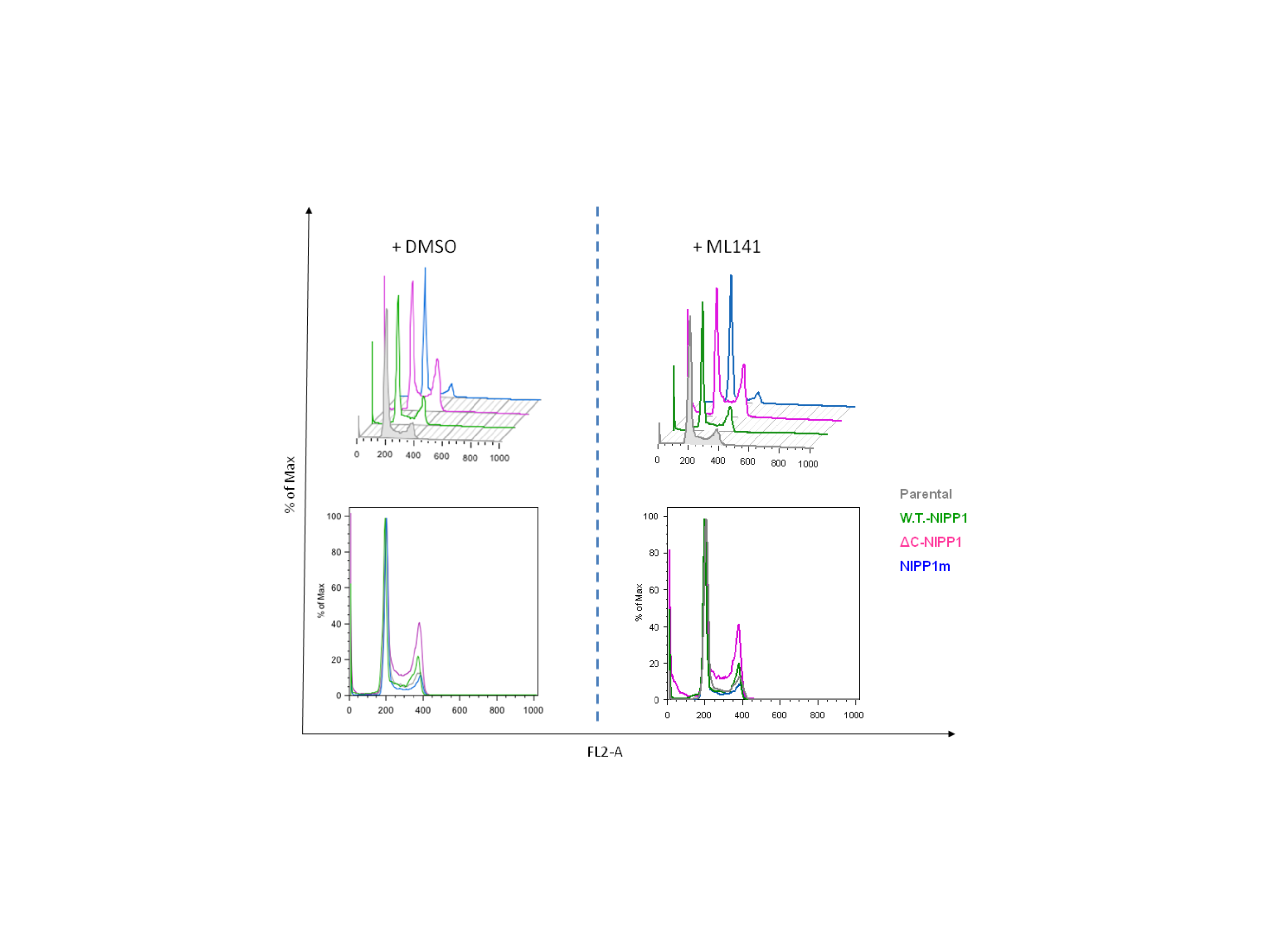

Supplement: Figure S1 — FACS analysis showing the effect of ML141 in the cell cycle of parental, W.T-NIPP1, ΔC-NIPP1 and mNIPP1 HeLa Tet-Off cells. ML141 (1 h pre-treatment) does not have an effect on cell cycle of parental, W.T-NIPP1 and mNIPP1 cells, however the sub-G1 population of ΔC-NIPP1 cells appears increased. Three experiments were performed with similar results and a representative experiment is shown. (TIF) [file pone.0040769.s001.tif]
